# Research on motion control of a novel stroke rehabilitation robot based on the dual parallel washout algorithm

**DOI:** 10.3389/fbioe.2025.1704300

**Published:** 2026-01-05

**Authors:** Junjie Yao, Zhongxu Li, Cuiping Wang, Junyu Wu, Yubin Liu, Deyu Cong, Huijuan Lou

**Affiliations:** 1 College of Acupuncture and Tuina, Changchun University of Chinese Medicine, Changchun, Jilin, China; 2 Department of Tuina, The Affiliated Hospital to Changchun University of Chinese Medicine, Changchun, Jilin, China; 3 State Key Laboratory of Robot Technology and Systems, Harbin Institute of Technology, Harbin, Heilongjiang, China

**Keywords:** redundant actuation, stroke rehabilitation robot, multi-source heterogeneous information fusion, multi-biosensor systems, dual parallel washout algorithm

## Abstract

A motion control strategy based on multi-source heterogeneous motion information fusion and motion decoupling parallel washout algorithm (WA) is proposed for the control of a rehabilitation robot designed for stroke-related balance disorders. The robot features a serial-parallel hybrid structure and humanoid gait functionality, with its output being the pre-defined trajectory motion of the guiding pedals. The WA algorithm is widely applied in motion simulation and control. In this study, the filter parameters of the WA are optimized using Multi-Objective Genetic Algorithm (MOGA), aiming to minimize the motion perception error introduced by the robot, thereby optimizing the robot’s motion trajectory to better align with the human perception threshold and the dynamic response characteristics of the device. A custom-built multi-source heterogeneous sensing system is employed to capture human gait features, enabling the WA to generate specific motion trajectories pre-defined for the rehabilitation robot. To ensure that the optimization search space for each WA channel remains independent and to more accurately reproduce motion details, motion decoupling and dual parallel filtering control strategies are introduced. Through the optimization of the WA filter parameters, the system aims to minimize the theoretical motion perception error experienced by the user during robot-assisted motion training, with the potential to provide a more realistic motion experience and enhanced training outcomes. In the future, long-term follow-up and monitoring of the effectiveness will also be conducted.

## 1 Introduction

**FIGURE 1:**
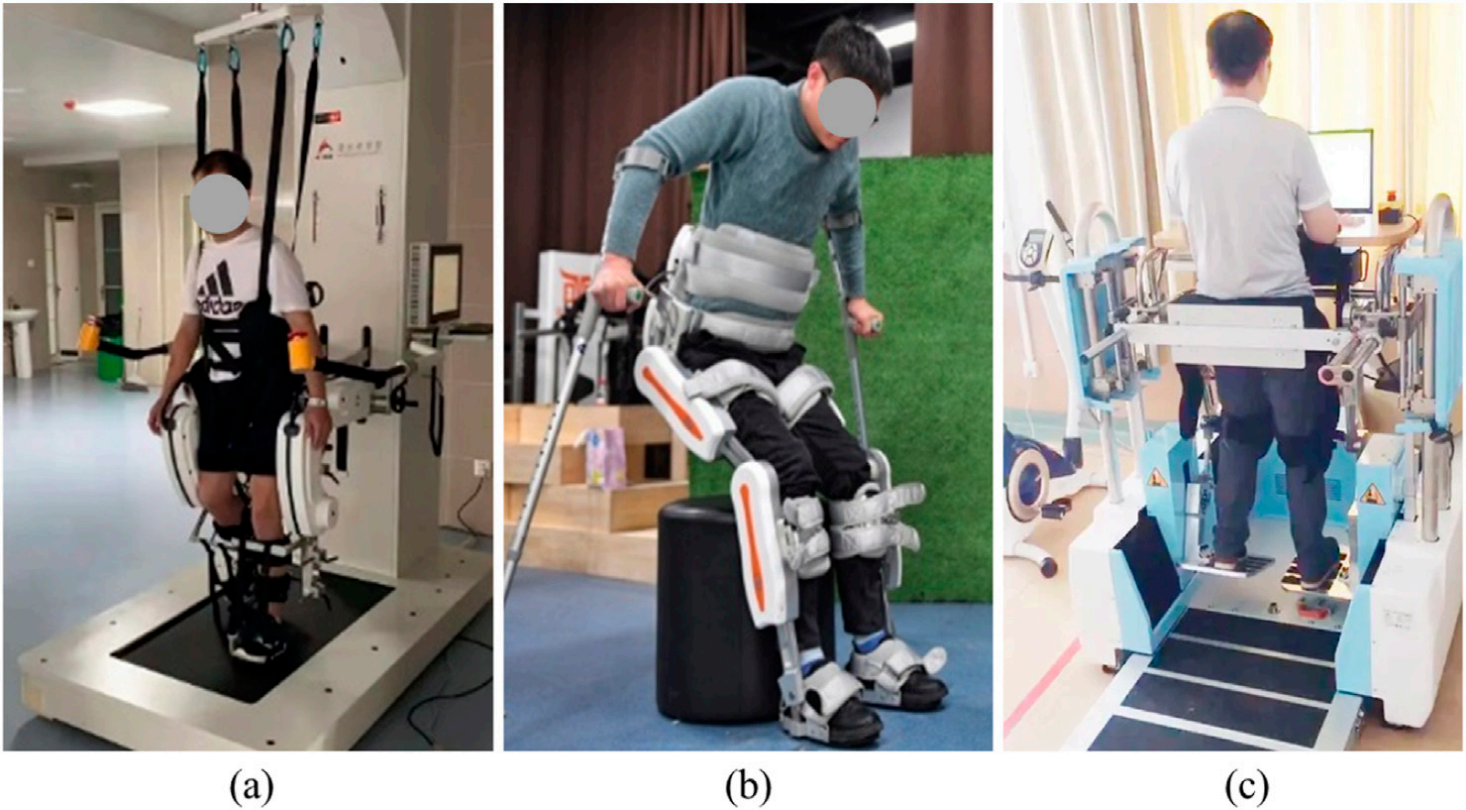
Typical rehabilitation training robots. **(a, b)** Exoskeleton robots; **(c)** End-effector robot.

With the increasingly serious aging problem and the rising number of stroke patients, the number of rehabilitation hospitals and departments has been growing annually, accompanied by a surge in demand for rehabilitation services. Insufficient treatment resources, the low efficiency of traditional rehabilitation therapy, and advancements in rehabilitation medicine and technology are driving the emergence and continuous development of rehabilitation robots. Rehabilitation robots represent the largest proportion within the classification of medical robots and are widely employed in clinical rehabilitation training, such as in the recovery treatment of stroke patients. The development of rehabilitation robots encompasses humanoid gait lower limb rehabilitation robots, lower limb exoskeleton robots, and treadmill-based lower limb rehabilitation robots (Zhang Y. et al., 2024; Chen et al., 2022). These robots are capable of performing repetitive exercise training and supporting multiple exercise modes, thereby promoting neural plasticity and improving patients’ motor function. Some advanced rehabilitation robots can collect and analyze data during the rehabilitation process, providing objective evaluation metrics for therapists and assisting physicians in formulating personalized rehabilitation prescriptions (Castelli et al., 2023; Lee et al., 2020; Sobiech et al., 2022). Figure 1 illustrates typical rehabilitation training robots. Figure 1a shows a treadmill-based rehabilitation robot, Figure 1b presents an exoskeleton robot, and Figure 1c depicts a pedal-type rehabilitation robot.

The motion control of redundant robots involves the planning and execution of movements in robotic systems with redundant Degree of Freedom (DOFs) to perform specific tasks and actions. Redundant robots possess more than the minimum DOFs necessary to complete tasks, offering greater flexibility in motion. This redundancy enables the robot to navigate obstacles, avoid singularities, and optimize specific performance indicators, enhancing the robot’s adaptability and robustness. However, redundant robots may present multiple kinematic solutions, necessitating the careful allocation of force and motion. Moreover, their control algorithms are often complex and computationally intensive, which poses significant challenges in the effective control of these robots. Common motion control techniques for redundant robots include the optimization of various performance indicators, such as minimizing motion time, energy consumption, and joint wear, by leveraging the redundant DOFs; fitting robot motion or dynamic models through intelligent control algorithms based on large-scale data, such as neural networks; and coordinating motion and force (Zhang X. et al., 2024; Lee et al., 2024; Conti, 2023).

**FIGURE 2:**
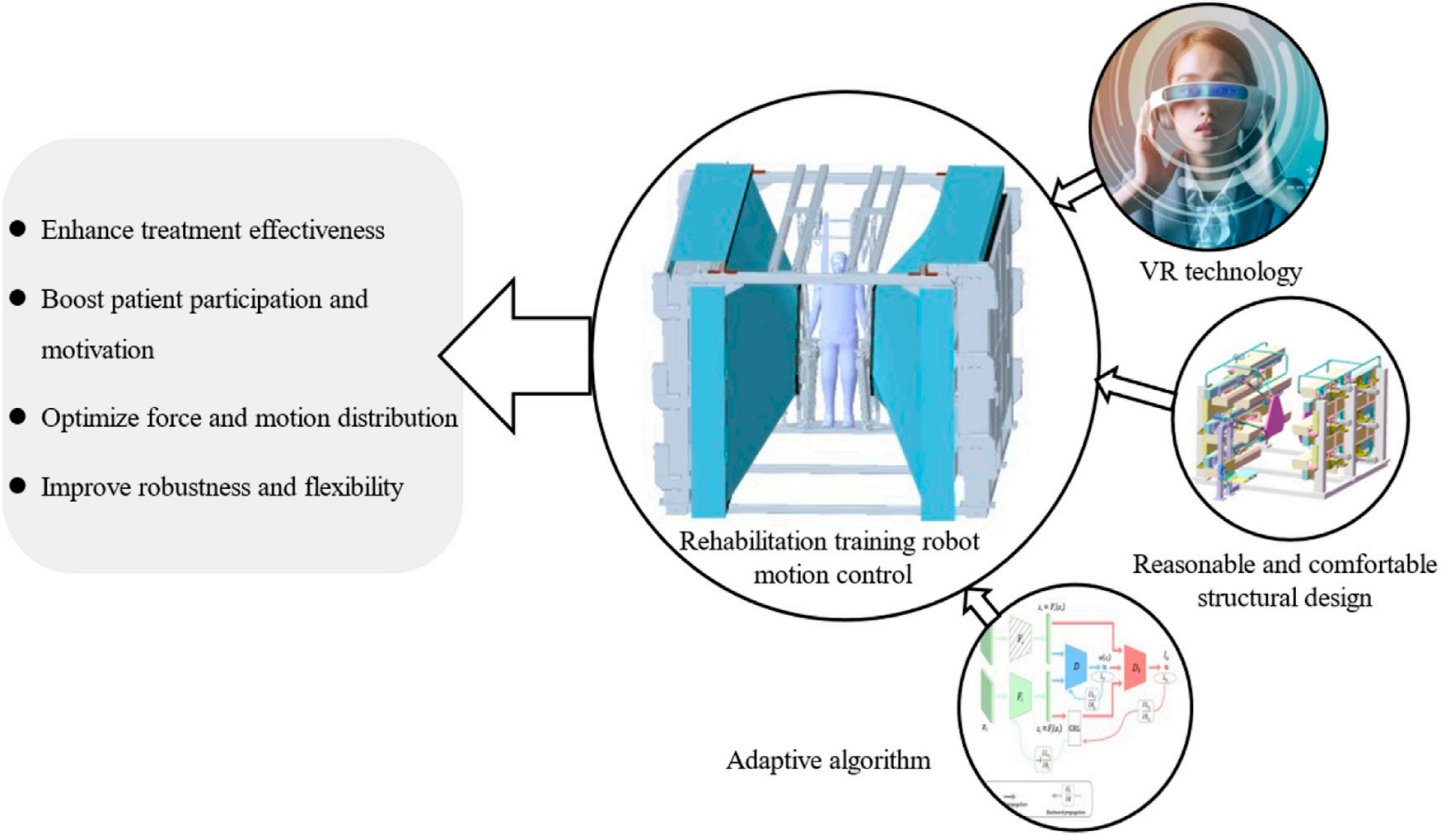
Motion control method and significance of redundant robots.

Physical perception plays a critical role in rehabilitation robots. A high-quality physical perception enhances user immersion in rehabilitation training, thereby increasing their participation and enthusiasm for treatment. By simulating realistic movement sensations, rehabilitation robots can better stimulate the user’s nervous system and muscles, thereby promoting functional recovery. To enhance the somatosensory realism provided by rehabilitation robots, techniques such as integrating virtual reality technology to create an immersive rehabilitation environment can be employed. Additionally, sensory experiences can be further enriched through visual, auditory, and other sensory stimuli. The structural design of rehabilitation robots takes user comfort into account, minimizing additional discomfort during rehabilitation and promoting more spontaneous movement. Adaptive algorithms are also incorporated to adjust the robot’s movements according to the patient’s specific movement patterns, ensuring that the robot’s actions are better aligned with the actual needs of the patient (Qu et al. (2017); Bergholz et al. (2023)). Figure 2 illustrates the motion control methods and significance of redundant robots.

The washout algorithm (WA) is a crucial component in simulating human body sensations across various motion simulators, such as airplane and car simulators. In certain motion simulations, a primary challenge is the limited motion space and travel of the simulation platform. Many real-world movements, such as human walking, are typically continuous in one direction, and the WA enables the simulation platform to realistically replicate these movements within a constrained range of motion. The WA takes advantage of the limitations of the human motion perception system. When linear acceleration or angular velocity falls below the human perception threshold, individuals are unable to detect the corresponding stimuli. The WA allows the simulation platform to execute specific movements without the user perceiving them, thus providing continuous motion sensory input. Initially used in flight simulators, the WA ensures that aircraft motion is simulated within a limited space while preventing motion beyond the human perception threshold. In rehabilitation training, this algorithm can assist rehabilitation equipment in simulating human movements more realistically, thereby theoretically improving the efficacy of rehabilitation training (Zhang and Xu, 2017; Dujeancourt and Garz, 2023; Nguyen and Hekman, 2024; Meeseub et al., 2010).

This study focuses on a motion control strategy for a redundantly driven rehabilitation training robot. The subsequent research content includes: 1. Introduction to the robot, 2. The WA and its optimization, 3. Optimization and application of the motion-decoupled dual parallel WA, 4. Experimentation and summary.

## 2 Materials and methods

### 2.1 The rehabilitation robot

**FIGURE 3:**
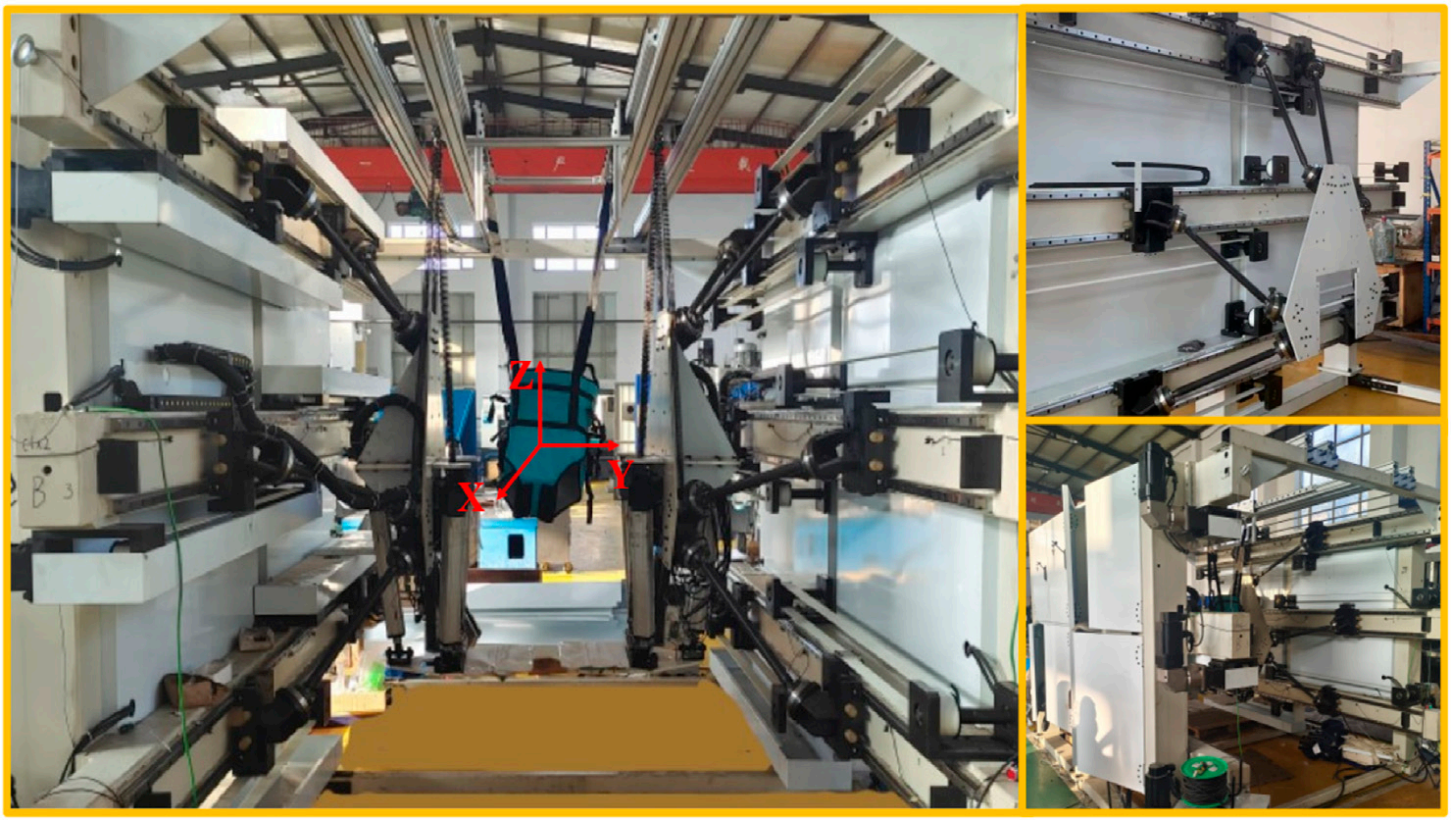
The physical image of the rehabilitation training robot.

In previous studies, a rehabilitation training robot with a serial parallel hybrid structure and redundant actuation, which is a lower limb end rehabilitation robot, was proposed; it contains two motion pedals that can simulate human gait and support and assist users in completing exercise training. The physical image and coordinate system definitions of the rehabilitation training robot are shown in Figure 3. The rehabilitation robot is composed of two sets of mirror-arranged devices that work together to complete the task. Each set of devices is composed of a 6 DOF parallel device and a 3 DOF parallel device and is referred to as the 6-SSP device and the 3-RPS device, respectively, based on the characteristics of their motion branches. The 3-RPS device is an extended device introduced by optimizing and improving the 6-SSP device. As a redundant mechanism, it can increase the working space, improve the mechanical performance of the 6-SSP device, and relieve the load pressure of the 6-SSP device during rotation. In previous studies, kinematic and dynamic modeling analyses of a robot, as well as the allocation principles of DOFs, were performed. Based on the results of motion and mechanics analysis, the 6-SSP device mainly completes linear motion, whereas the 3-RPS device completes rotational motion, thus leveraging the advantages of both devices, optimizing the load distribution during motion, and improving the overall performance (Wu et al. (2023); Wu et al. (2022); Wu et al. (2024)).

### 2.2 Multi-biosignal motion sensing system

In order to extract gait features of human motion, this study developed a custom multi-source heterogeneous biological motion information sensing system. The system comprises primarily displacement sensors, Inertial Measurement Units (IMUs), and pressure sensors. The displacement sensors and IMUs are used to capture the displacement and posture changes of the ankle during movement, while the pressure sensors measure the interaction forces between the foot sole and the environment. The motion data obtained from this sensing system undergoes preprocessing and multi-source heterogeneous information fusion to generate raw motion data. The processed data is then passed through the Washout Algorithm (WA) to generate predefined trajectories for the robot. The robot subsequently assists and guides patients with stroke in performing designated rehabilitation exercises, thereby aiding in the therapeutic process.

### 2.3 The WA and its optimization

#### 2.3.1 Application and significance of the washout algorithm in rehabilitation

**FIGURE 4:**
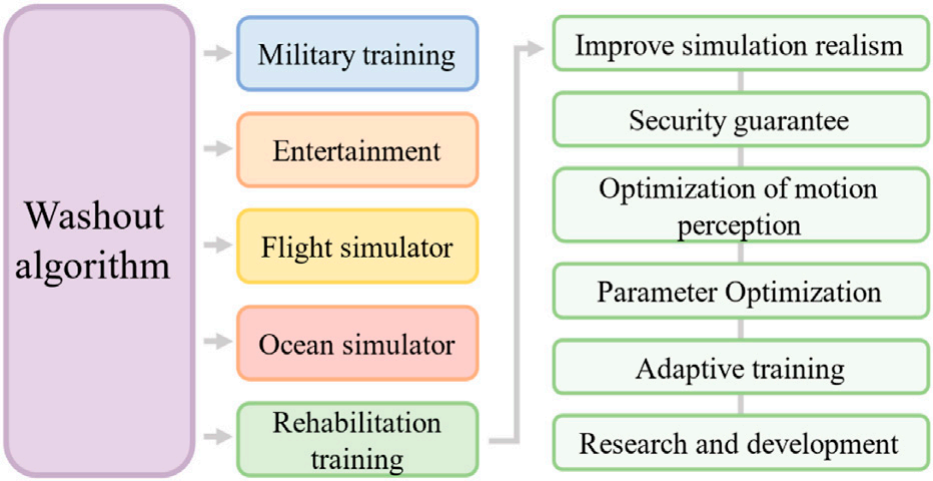
The application and significance of the WA in rehabilitation.

In the field of rehabilitation training, WAs can ensure that rehabilitation equipment simulates human movements more smoothly and naturally improves the realism of training. By adjusting the parameters of the algorithm, personalized training can be provided for different patients’ rehabilitation needs and exercising abilities. The WA can control the range of motion of rehabilitation equipment, ensuring that the movement does not exceed the safety limit and protecting patients from injury. This algorithm can optimize patients’ perception of movement, helping them better understand their own movement status and rehabilitation progress. By using genetic algorithms or other optimization techniques, the optimal WA parameters can be found to improve the performance of rehabilitation equipment. The application of the WA in rehabilitation training has promoted the research and development of related equipment and advanced rehabilitation technology. Figure 4 illustrates the potential functionality and value of the WA in rehabilitation training.

#### 2.3.2 Principle of tilt coordination

The human perceives motion speed through vision and perceives linear acceleration and angular velocity through the vestibular organs. Among them, otoliths can sense linear acceleration, and semicircular canals can sense angular velocity. Introducing the mathematical models for the otolith and semicircular canals, the transfer function (Laplace transform) for the otolith is represented by Equation 1, and the transfer function for the semicircular canals is represented by Equation 2.

$$G_{\mathrm{oto}}\left(s\right)=\frac{k\cdot \left(\tau_{a}\cdot s+1\right)}{\left(\tau_{l}\cdot s+1\right)\left(\tau_{s}\cdot s+1\right)}$$ (1)

$$G_{SC}\left(s\right)=\frac{T_{a}\cdot T_{l}\cdot s^{2}}{\left(T_{l}\cdot s+1\right)\left(T_{s}\cdot s+1\right)\left(T_{a}\cdot s+1\right)}$$ (2)

**FIGURE 5:**
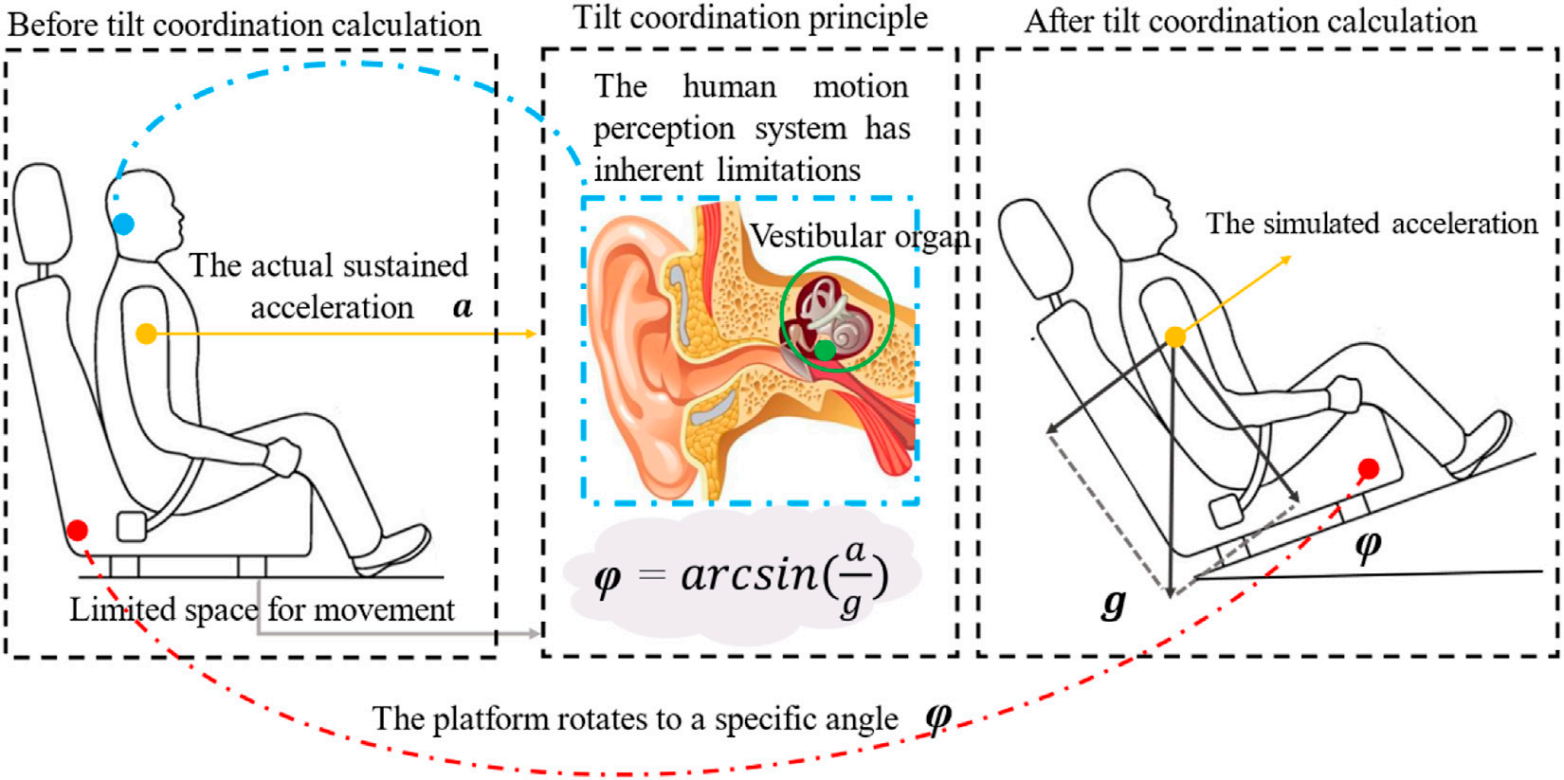
Tilt coordination.

$\tau_{a}$ , $\tau_{l}$ , and $\tau_{s}$ represent the time constant for the otolith, the long time constant, and the short time constant, respectively. $T_{a}$ , $T_{l}$ , and $T_{s}$ represent the time constant for the otolith, the long time constant, and the short time constant, respectively. In the process of simulating motion in simulators, it is often necessary to simulate continuous acceleration. However, owing to the limited workspace and range of motion of the simulator, the motion platform cannot directly provide continuous acceleration. To achieve sustained acceleration sensation in simulators, it is necessary to utilize the deficiencies in human perception. The vestibular organs of the human body cannot perceive whether the specific force is generated by linear acceleration or the component of gravity. Therefore, the platform can be rotated at a certain angle to generate the specific force that the human body can perceive by the component of gravity in the direction of motion (Wang and Zhang, 2019; Meeseub et al., 2010; Li et al., 2025). At this point, the platform may not provide this portion of linear acceleration. The rotation of the platform is an additional motion generated to simulate linear acceleration and should not be perceived by the human body. The angular velocity of the platform rotation should be less than the human perception threshold. The threshold for human motion perception is shown in Table 1A schematic diagram of the tilt coordination is shown in Figure 5.

**TABLE 1:** The threshold for human motion perception.

Motion signal	Perception threshold
Lateral acceleration	0.17 m/s^2^
Longitudinal acceleration	0.17 m/s^2^
Vertical acceleration	0.28 m/s^2^
Rolling angular velocity	3 ◦ /s (0.05236 rad/s)
Pitch angular velocity	3.6 ◦ /s (0.06283 rad/s)
Yaw angular velocity	2.6 ◦ /s (0.04537 rad/s)

**FIGURE 6:**
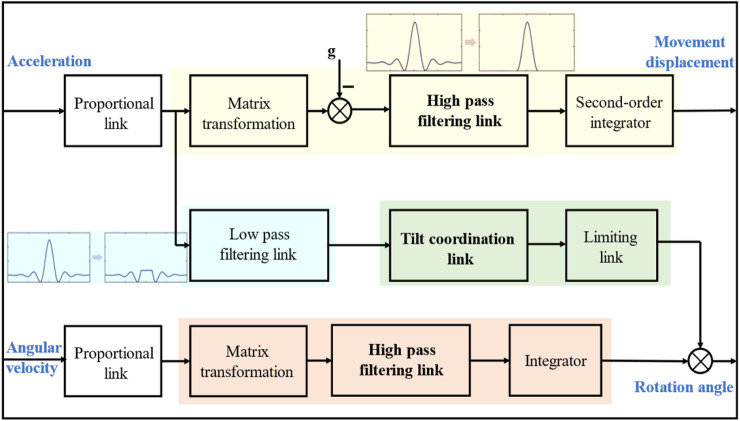
The framework of washout algorithm.

The WA consists of a series of filters, which primarily consists of four parts: the tilt coordination part, the coordinate transformation part, the acceleration high-pass part, and the angular velocity high-pass part. The role of the filters in each part is to convert the true motion (the motion expected to be perceived by the human body) into the actual motion of the simulator motion platform. The composition of the WA is shown in Figure 6.

The tilt coordination component consists of a second-order filter, is shown in Equation 3:

$$G\left(s\right)=\frac{s^{2}}{s^{2}+2\xi \omega s+\omega^{2}}$$ (3)

The other parts are third-order filters, and the general expression is given by Equation 4:

$$G\left(s\right)=\frac{s^{2}}{s^{2}+2\xi \omega s+\omega^{2}}\cdot \frac{s}{s+\omega^{\prime }}\;$$ (4)

The main parameters in the equation are the cutoff frequency ω and the damping ratio ξ .

In previous studies, a MOGA was employed to optimize the washout algorithm (WA) of a 6-DOF rehabilitation training robot. This 6-DOF rehabilitation robot represents the initial design version of the redundant rehabilitation robot in this research, which closely resembles the 6-SSP device. Henceforth, it will be referred to as the initial version (IV) robot (Wu et al., 2022). The filter parameters of the WA often vary depending on the specific movement. In earlier work, the lower limb acceleration signal during human motion was simplified into a periodic square wave signal, which was used as the expected motion command to identify the optimal filter parameters for somatosensory simulation. However, the motion characteristics in this simplified signal were not well-defined, and some critical details were lost, potentially leading to inaccuracies in the optimization results. Therefore, in this research, real gait data from human walking motion were collected. The otolith model of the human body was introduced to generate the ideal acceleration signal. The acceleration signal produced by the platform’s movement, as perceived by the otolith, represents the true acceleration, with the difference between the two signals corresponding to the human-perceived acceleration error. Similarly, the semicircular canal model was applied to determine the human angular velocity error. In the previous research and optimization of control algorithms, several primary objectives were identified. One goal was to minimize the displacement and rotation angle of the simulated platform, given the limited mechanical characteristics of the IV robot, which has a smaller motion stroke. Another key objective was to minimize somatosensory errors for the user, including acceleration somatosensory error (ASE) and angular velocity somatosensory error (AVSE). As a result, four optimization objectives were considered, with the design variables being the filter parameters (Wu et al., 2022). However, the impact of the design variables on the two objectives—somatosensory error and simulation platform motion range—was antagonistic. Therefore, it was necessary to assign weights to these objectives based on their relative importance and calculate the design variable values that would optimize the objectives through a compromise approach.

### 2.4 Optimization and application of a dual parallel washout algorithm based on motion decoupling

#### 2.4.1 Establishment of WA optimization model and research on motion control

The redundant robot possesses more DOFs than the six required for achieving spatial global positioning. To ensure that each DOF can be independently controlled, allowing each joint or control unit to move without interfering with one another, it is essential to devise a motion decoupling control strategy. Previous studies have demonstrated that the 6-SSP device excels in linear motion, while the 3-RPS device is particularly effective in rotational motion. The advantage of these two devices lies in their complementary nature, whereby they work together to determine the allocation of DOFs between them (Wu et al., 2023). The principle of decoupled motion control enhances task allocation and execution, making it more flexible and efficient. Following decoupling, the path of each joint can be planned independently, improving the efficiency and flexibility of path planning. Decoupling also simplifies control algorithm design, as each DOF can be treated separately, resulting in a more intuitive and easier-to-implement control strategy. In the context of the optimization problem for the WA (Workspace Allocation) of the IV robot, it is expected that motion instructions will encompass both linear and rotational motion information. The robot’s motion range and the motion error in the optimization objective are mutually constrained. The limited motion range of the robot and the insufficient reproduction of ideal motion will inevitably degrade the realism of the motion. In research on IV robots, different optimization objectives are assigned varying weights, with more important objectives receiving higher weights. During the optimization process, design variables are computed in favor of objectives with higher weights, which may prevent a balanced optimization of both objectives. The structure of the “6-SSP + 3-RPS” redundant robot is modular, with the two devices contributing to the movement of different DOFs according to their respective advantages. The 3-RPS device, as a supplementary redundant device, provides additional DOFs, improving fault tolerance and offering more options for path and attitude when performing tasks. Furthermore, it can redistribute task loads, alleviating the rotational pressure on the 6-SSP device, thus enhancing the mechanical properties of the robot and expanding its workspace. This results in a significant improvement in the originally constrained and limited workspace. In previous optimizations of the IV robot, the robot’s limited workspace required a reduction in its range of motion to avoid exceeding its working limits. However, reducing the robot’s motion amplitude also led to insufficient motion simulation, restricting the alleviation of motion errors. For this redundant robot, the workspace margin during rehabilitation training is sufficiently large, allowing the motion amplitude of the platform to be disregarded during optimization. The 6-SSP device primarily performs high-speed and high-acceleration linear motion, with its WA converting high-pass acceleration signals into linear motion for the platform. In optimizing the simulated motion, the foremost consideration is the real acceleration sensation induced by the device’s motion. The 3-RPS device facilitates flexible rotational motion, and in the optimization process, the rotational limit positions of the platform can be disregarded. The primary focus is on the angular velocity sensation resulting from the device’s motion. In human walking and other movements, the coordinated work of lower limb translation and rotation is required to perceive and control body movement. Translational motion mainly enables the perception of body position in space and facilitates body propulsion, while rotational motion primarily supports gait adjustment and influences body sensation, contributing to the perception of the body’s motion state. Therefore, for redundant robot devices, the optimization and application of the WA for the entire system will no longer be conducted as a unified process. Instead, the two modular devices will be treated separately, optimized, and applied individually. The 6-SSP device will function as a linear motion module, with the expected motion command also being linear following motion decomposition. The optimization objective of the WA is motion ASE, as outlined in Equation 5.

$$minimize\left\{ASE\right\}=\;minimize\left\{L\left[a\left(t\right)\right]\times \left(G_{\mathrm{oto}}\left(s\right)-G_{\mathrm{oto}}\left(s\right)\times G_{ah}\left(s\right)\right)\right\}$$ (5)

In the equation, L[⋅] represents the Laplace transform, a(t) is the time-domain acceleration signal, and $G_{ah}\left(s\right)$ is the high-pass acceleration transfer function. The expected motion command for the 3-RPS device that completes rotational motion is the rotational part after motion decomposition. The optimization goal of the WA is to reduce the motion AVSE and provide a realistic level of motion simulation. Formula 6 presents the optimization objective of the WA for the 6-SSP device.

$$minimize\left\{AVSE\right\}=minimize\left\{L\left[\omega \left(t\right)\right]\times \left(G_{cs}\left(s\right)-G_{cs}\left(s\right)\times G_{\omega h}\left(s\right)\right)\right\}$$ (6)

**FIGURE 7:**
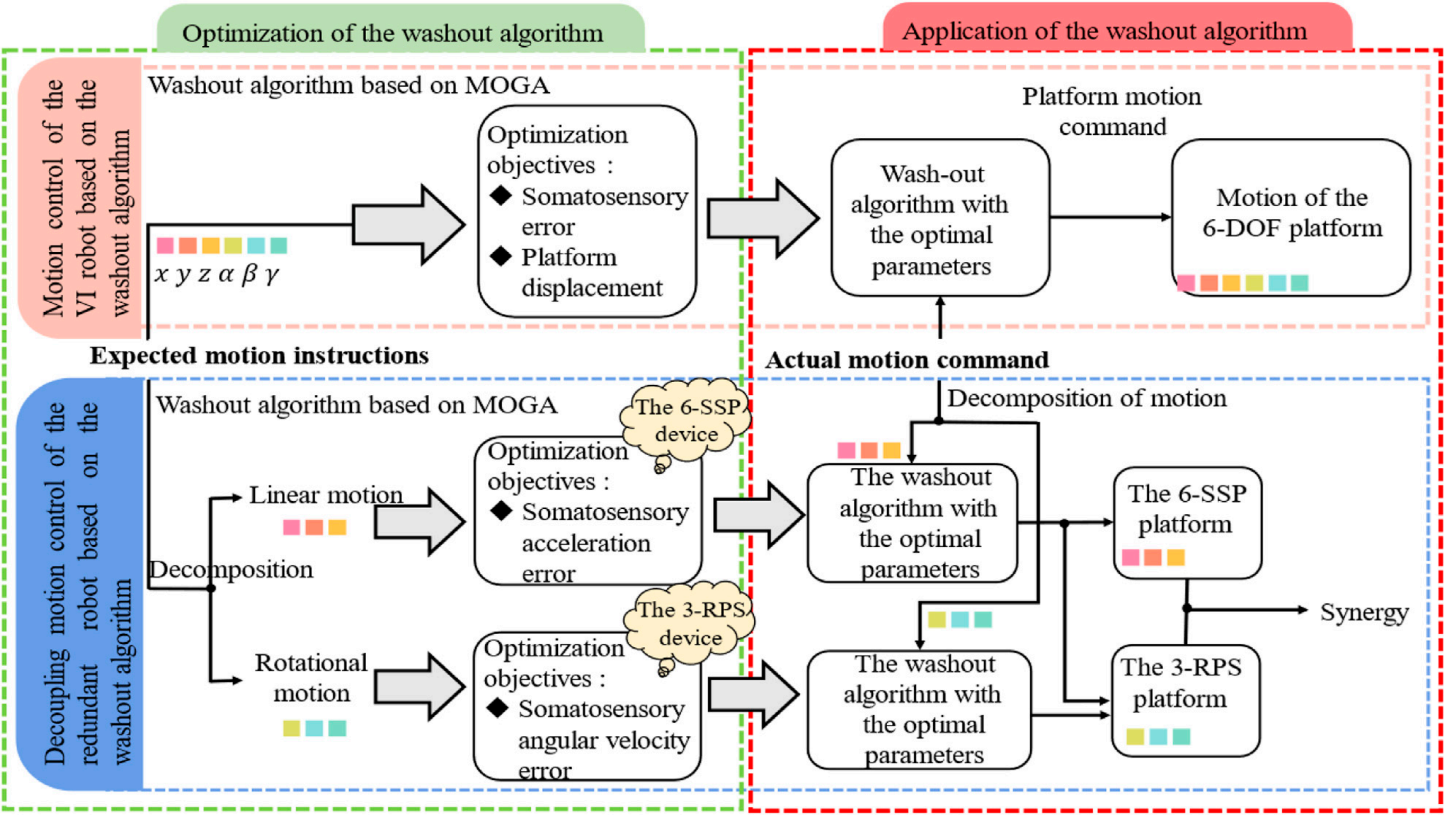
Optimization and application control block diagram of the IV robot and redundant robot washing algorithm.

Therefore, for the whole machine, two sets of washing algorithms are parallel, and since both the devices perform their own duties, the optimization objectives of their corresponding washing algorithms are also different. Each WA has only one objective, avoiding the problem of design variables being overly biased toward one objective. Moreover, both objectives are balanced and equally emphasized, which improves the overall motion control performance of the machine and reduces the computational complexity and workload. Figure 7 compares the IV robot and the redundant robot in terms of motion control.

#### 2.4.2 Simulation study on the optimization and application of the washout algorithm

**FIGURE 8:**
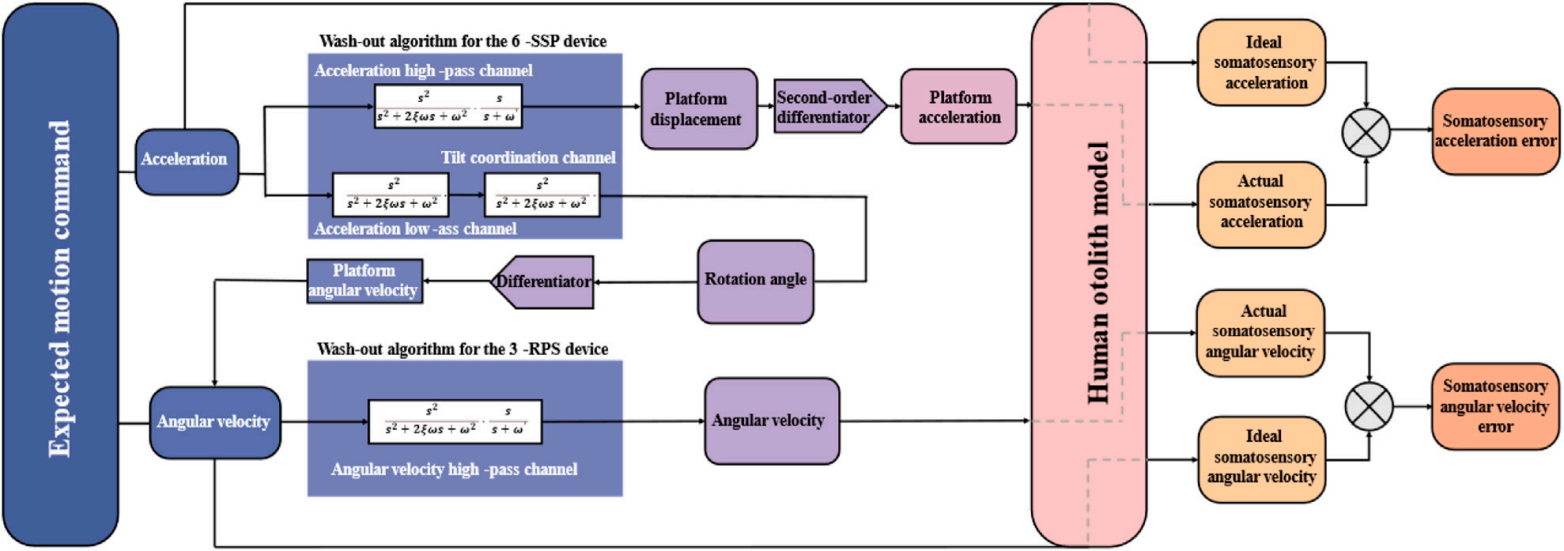
The optimization and application process of the WA.

Due to the variation in filter parameters for different motions, an empirical parameter may not be universally applicable across all application scenarios. In the context of rehabilitation training, the redundant robot aids users in completing walking motion tasks. Therefore, for this specific application scenario, it is essential to identify the filter parameters that result in the highest motion realism and optimal motion simulation. Volunteers wore motion data collection devices to perform walking movements and gather gait data during walking. This self-constructed walking gait dataset is then used as input to optimize the WA. For the 6-SSP device, the input for optimizing its WA consists of the linear portion of the collected gait data. The MOGA, which has global search capabilities, is employed to determine the optimal filter parameters, thereby achieving a more realistic acceleration motion sensation for the optimized 6-SSP device. The WA with the best parameters is subsequently applied to control the motion of the 6-SSP device. In accordance with the DOF decomposition principle, when controlling the 6-SSP device’s motion, only the linear data from the actual motion command is input. This data is then transformed into the platform’s motion information through the WA, comprising both linear and rotational motion components. The linear motion component is executed by the 6-SSP device itself, while the rotational motion component is handled by the 3-RPS device. For the 3-RPS device, the input for optimizing its WA includes the rotational data portion of the collected gait data, and the optimization objective is to minimize the AVSE. The MOGA algorithm is utilized to identify the filter parameters that correspond to the minimum sensory error during motion. Once the WA with the optimal filter parameters is applied to the 3-RPS device’s motion control, only the rotational data part of the actual motion command is input, which is then transformed into rotational motion information for the platform through the WA and executed by the 3-RPS device. Figure 8 illustrates the optimization and application process of the WA for this redundant robot.

**FIGURE 9:**
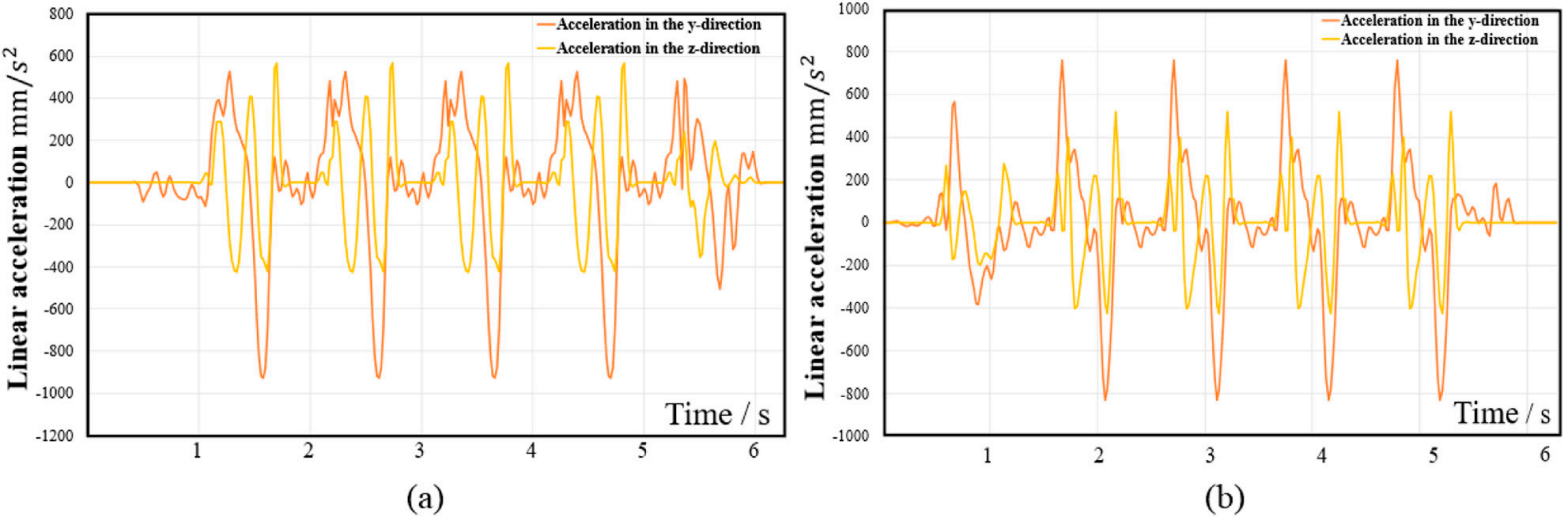
The acceleration information **(a)** left foot; **(b)** right foot.

**FIGURE 10:**
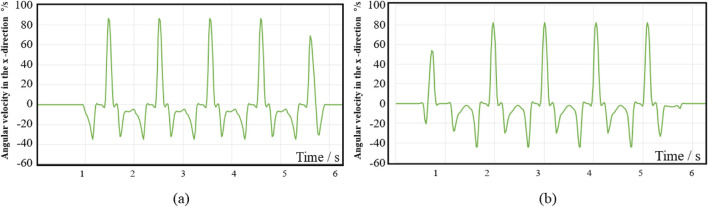
The collected motion angular velocity information. **(a)** left foot; **(b)** right foot.

In accordance with the principles of motion decomposition and optimization objectives, the MOGA is used to improve the WAs of the 6-SSP and 3-RPS devices, and the collected gait data is used as the expected motion commands for the algorithm application scenario. Figures 9, 10 show the motion gait data information, including four gait cycles of walking. Figure 9a shows the acceleration information of the volunteer’s left foot, Figure 9b shows the acceleration information of the volunteer’s right foot, Figure 10a shows the angular velocity information of the left foot, and Figure 10b shows the angular velocity information of the right foot.

**FIGURE 11:**
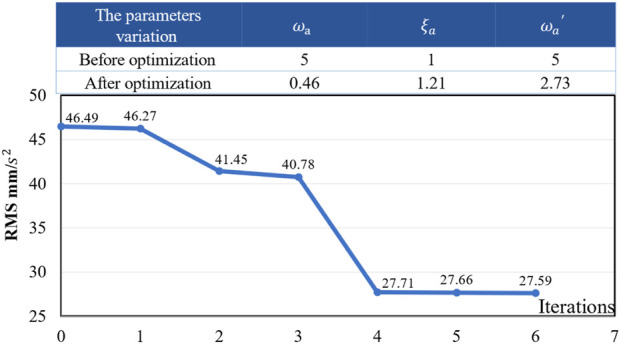
The iterative process of the objective function.

For the left 6-SSP device, the acceleration signal in the Y-direction is shown in Figure 9A, which is smoothed by sliding filtering and used as the expected motion command signal optimized by the WA. For the high-pass acceleration component in the Y-direction, the formula for its filter is shown in Formula 4 above, where the parameters include the cutoff frequency ω , $\omega^{\prime }$ and damping ratio ξ and are used as design variables for the MOGA. In the process of optimizing the WA parameters, the objective of the function is to minimize the root mean square of the ASE. In iterative optimization, the initial values of the design variables are taken as $\omega_{a}=5$ , $\omega_{a}^{\prime }=5$ and $\xi_{a}=1$ . Figure 11 shows the iterative process of the objective function and the parameters of the Y-direction acceleration high-pass filter before and after optimization. After 7 iterations, the optimization objective is minimized, and the design variables obtain the global optimal values. The optimal parameter values are $\omega_{a}=0.46$ , $\omega_{a}^{\prime }=2.73$ , and $\xi_{a}=1.21$ .

**FIGURE 12:**
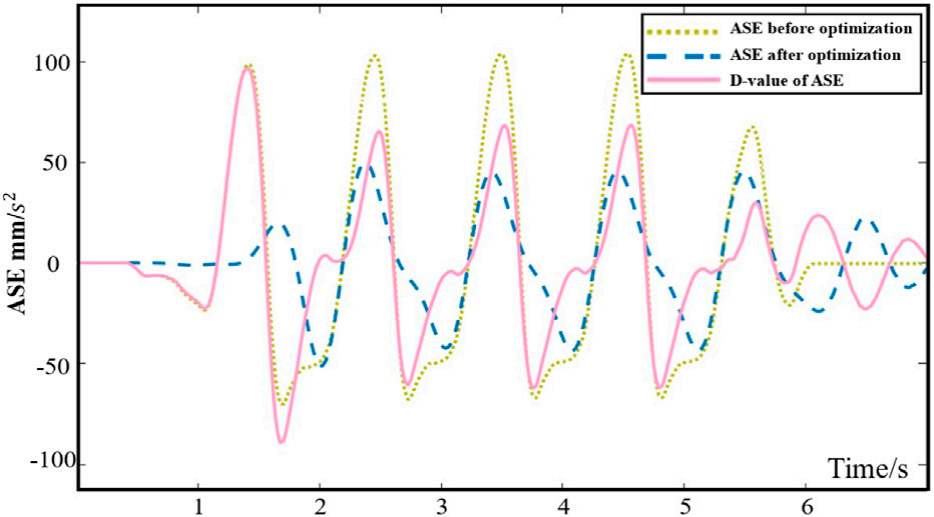
The Y-direction ASE before and after optimization.

Figure 12 before optimization, the amplitude of ABSE was 102 mm/s^2^, and after optimization, the amplitude of ABSE reduced to 68 mm/s^2^. The peak value of ABSE decreased by approximately 30% between the pre-optimization and post-optimization states. Multiple repetitions of the simulation process were conducted, and the results consistently showed stability and reliability. The optimal washout filtering parameters were ultimately determined, achieving a stable theoretical reduction of approximately 30% in the sensory error. After passing through the high-pass filtering channel for acceleration, the instantaneous high-frequency components of acceleration in the original signal were preserved, which was achieved through the motion of the 6-SSP device. The continuous low-frequency components of acceleration in the original signal were filtered out, and this portion was eventually compensated for by the gravity acceleration component through the inclination coordination channel. By utilizing the WA, the instantaneous high-amplitude acceleration signals were ultimately converted into the displacement of the 6-SSP device’s motion platform.

**FIGURE 13:**
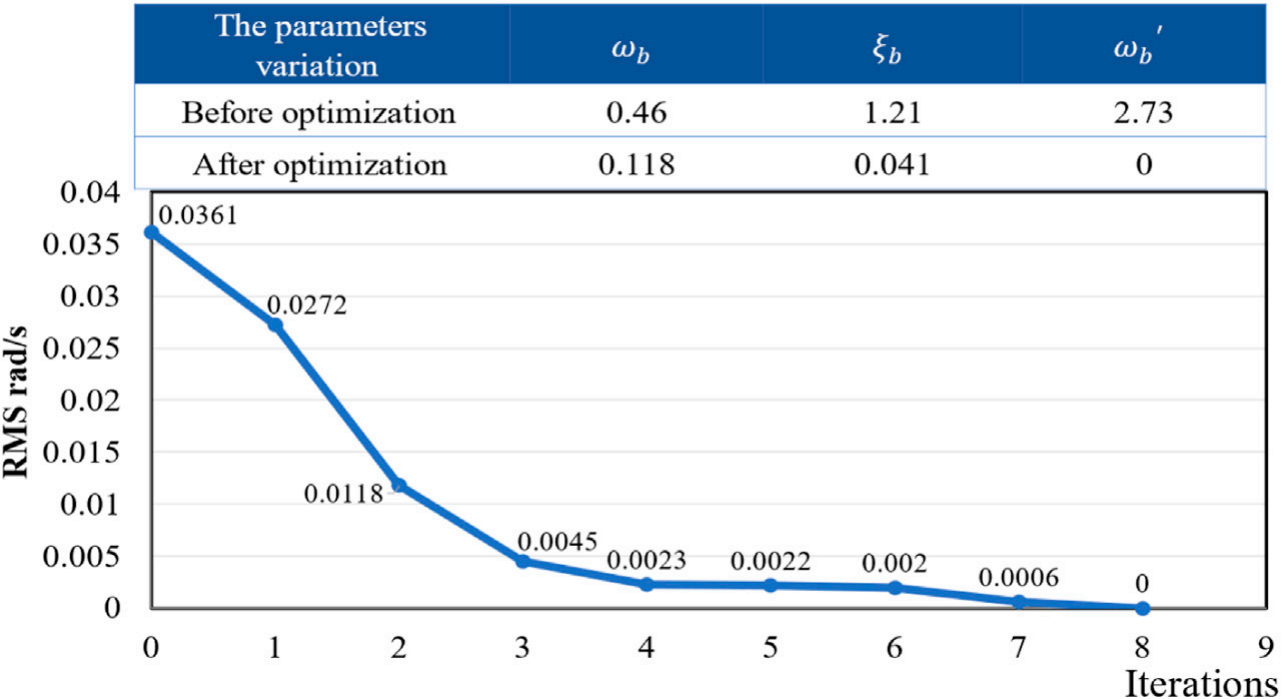
The iterative process of the objective function.

**FIGURE 14:**
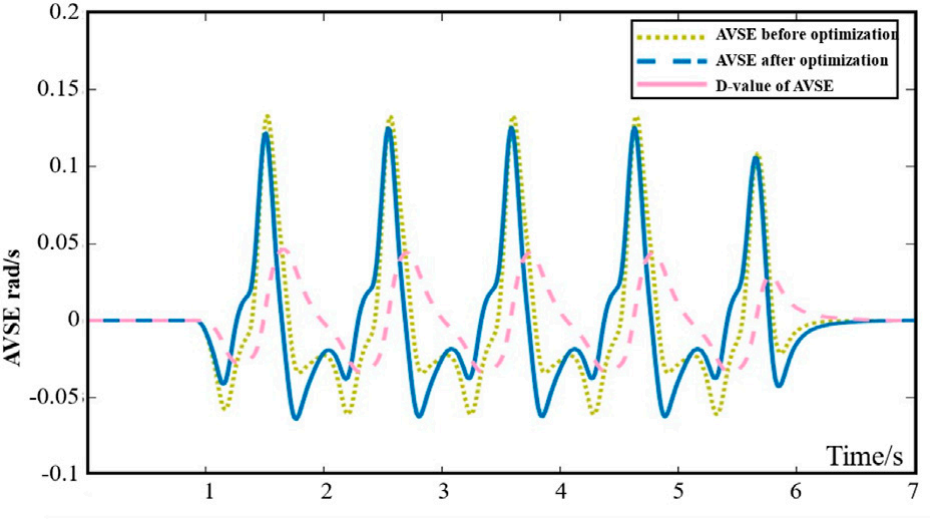
The AVSE before and after optimization.

The high-frequency component of the acceleration signal in the original motion command is finally set as the displacement of the 6-SSP device through the acceleration high-pass channel, while the remaining continuous low-frequency signal is finally converted into the rotation angle of the 3-RPS device through the tilt coordination channel. Therefore, for the 3-RPS device, the washout algorithm inputs two parts of motion commands during the optimization process: one part is the angular velocity part in the original motion command, and the other part is the output of the tilt coordination channel. Due to the fact that only the x-direction angle changes during human walking, only the x-direction angular velocity high-pass filter needs to be optimized. For the high-pass component of angular velocity in the X-direction, the formula for its filter is shown in the above Formula 4, where the design parameters are also $\omega_{b}$ , $\omega_{b}^{\prime }$ and damping ratio $\xi_{b}$ . In the process of optimizing the washout algorithm parameters, the objective function is to minimize the AVSE caused by the device motion. The initial values of the design variables in iterative optimization are based on the experience of the 6-SSP device washout algorithm, with $\omega_{b}=0.46$ , $\omega_{b}^{\prime }=2.73$ , and $\xi_{b}=1.21$ respectively. Figure 13 shows the iterative process of the objective function. After 8 iterations, the optimization objective is minimized, and the design variables achieve the global optimum. The optimal parameter values are $\omega_{b}=0.118$ , $\omega_{b}^{\prime }=0.041$ , and $\xi_{b}=0$ . Figure 14 shows the AVSE before and after optimization, and there is also a certain improvement in the angular velocity sensation caused by the device after optimization.

## 3 Results

**FIGURE 15:**
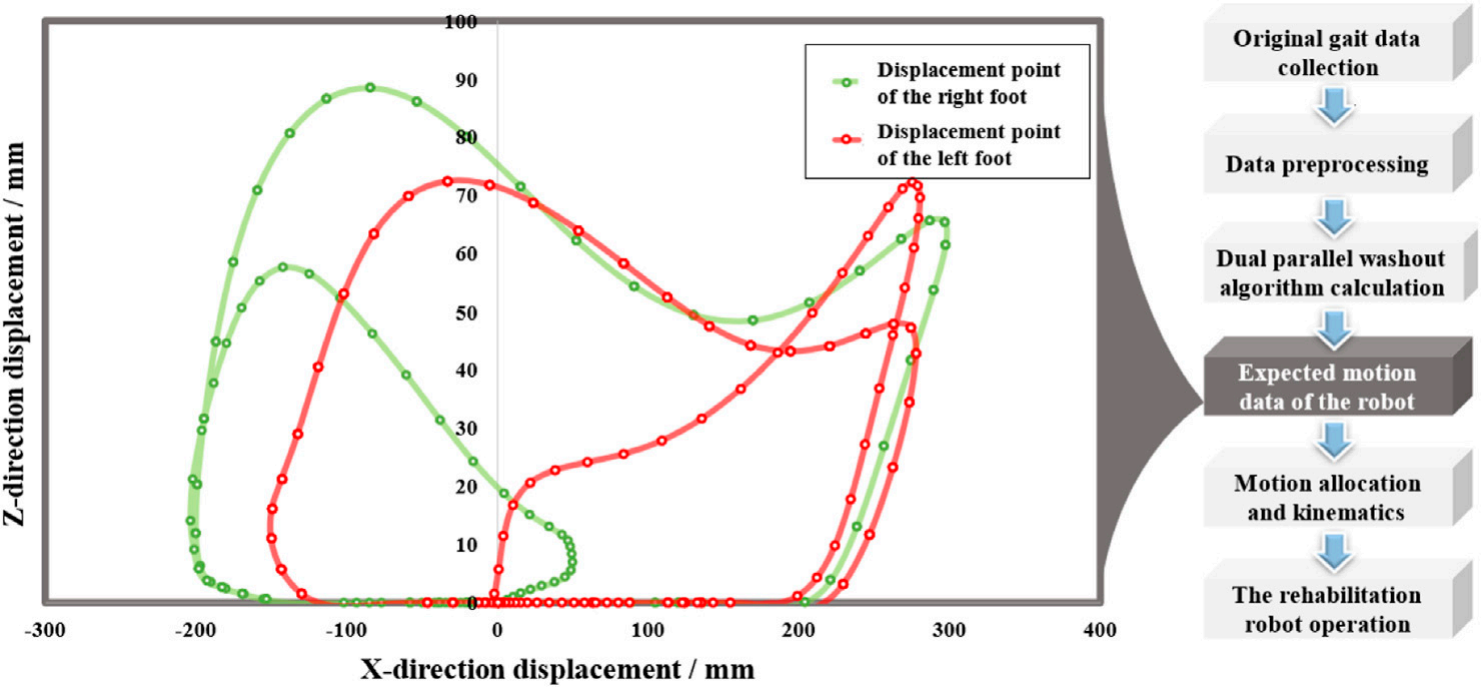
Motion trajectory in the X–Z plane.

**FIGURE 16:**
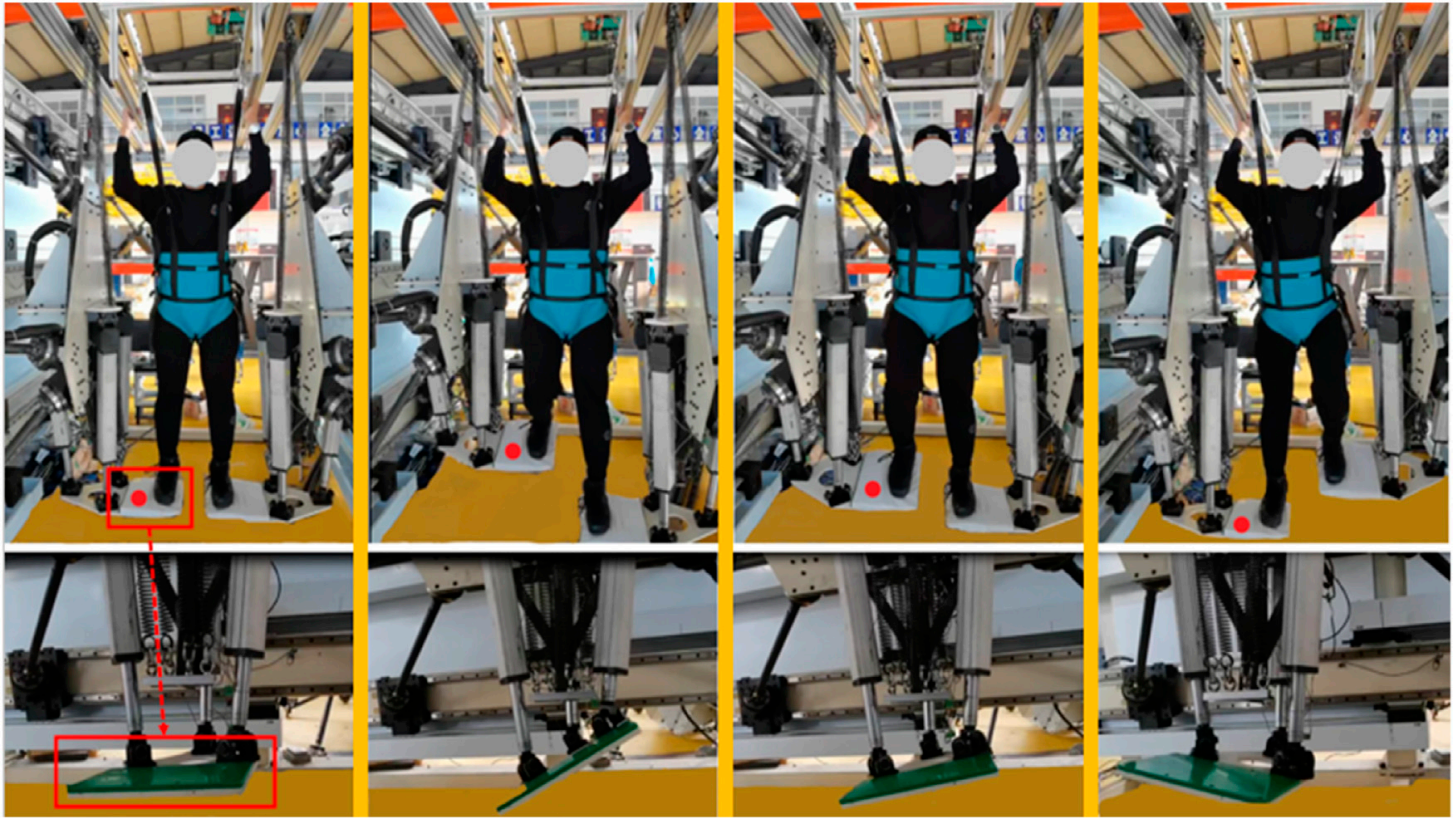
The situation of the actual robot during operation.

Analyzing the simulation results before and after optimization, for the 6-SSP device, when the parameter-optimized filter parameter WA is used, the realism of the perceived acceleration caused by device motion is greatly improved, and the device can better reproduce the acceleration information in motion commands. For the 3-RPS device, the angular velocity of the device before and after optimization did not exceed the required threshold (human perception threshold). By using the parameter-optimized filter WA, the AVSE was reduced, and the angular velocity simulation was improved. The optimized parameters comprehensively improved the performance of the platform motion simulation. The redundant robot is controlled to work according to the predefined motion trajectory after washout. The trajectory of the motion in the x‒z plane is shown in Figure 15. The continuous and large-scale walking motion data originally collected became limited in terms of periodic motion after being calculated by the WA. Figure 16 shows the situation of the actual robot during operation.

## 4 Discussion and conclusion

This study investigates a motion control algorithm for a redundant stroke rehabilitation training robot, which consists of a 6-SSP device and a 3-RPS device. A parallel motion decoupling control strategy based on the MOGA optimization WA is proposed. The 6-SSP device and 3-RPS device each utilize a distinct set of filter parameters with different WAs. The optimization objective of the 6-SSP device is to minimize the acceleration error induced by the device’s motion, while the goal of the 3-RPS device is to reduce the angular velocity error in motion perception, thereby enhancing the realism of the motion experience. These two optimization algorithms are handled separately and computed in parallel during control applications, ensuring that both performance criteria are satisfied. The issue of multiple conflicting objectives within a single optimization model is circumvented, and variables are designed to focus on specific optimization goals. A custom-built walking gait dataset, obtained through data collection, serves as the input motion instructions for WA optimization. In accordance with the principle of DOF decomposition established in previous research, the linear and rotational components of the motion instructions are allocated to the 6-SSP and 3-RPS devices, respectively, ensuring that the optimized WA aligns with the motion performance characteristics of each device. Simulation analysis results demonstrate that the proposed strategy effectively reduces perceptual error, thereby validating its theoretical effectiveness. Furthermore, its stability has been confirmed through human-machine experiments. However, the study does present certain limitations, as the rehabilitation efficacy requires long-term monitoring and evaluation. Future research will focus on further development of additional motion modes and conducting experiments with a wider range of participants.

## Data Availability

The raw data supporting the conclusions of this article will be made available by the authors, without undue reservation.

## References

[B1] Bergholz M. Ferle M. Weber B. M. (2023). The benefits of haptic feedback in robot assisted surgery and their moderators: a meta-analysis. Sci. Rep. 13, 19215. 10.1038/s41598-023-46641-8 37932393 PMC10628231

[B2] Castelli L. Iacovelli C. Ciccone S. Geracitano V. Loreti C. Fusco A. (2023). Robotic-assisted rehabilitation of lower limbs for orthopedic patients (roar-o): a randomized controlled trial. Appl. Sciences-Basel 13, 13208. 10.3390/app132413208

[B3] Chen W. Li J. Zhu S. Zhang X. Men Y. Wu H. (2022). Gait recognition for lower limb exoskeletons based on interactive information fusion. Appl. Bionics And Biomechanics 2022, 9933018. 10.1155/2022/9933018 35378794 PMC8976668

[B4] Conti S. (2023). How redundancy and distributed control are helping make robots autonomous. Nat. Rev. Phys. 5, 501. 10.1038/s42254-023-00636-6

[B5] Dujeancourt E. Garz M. (2023). The effects of algorithmic content selection on user engagement with news on Twitter. Inf. Soc. 39, 263–281. 10.1080/01972243.2023.2230471

[B6] Lee S. H. Park G. Cho D. Y. Kim H. Y. Lee J.-Y. Kim S. (2020). Comparisons between end-effector and exoskeleton rehabilitation robots regarding upper extremity function among chronic stroke patients with moderate-to-severe upper limb impairment. Sci. Rep. 10, 1806. 10.1038/s41598-020-58630-2 32019981 PMC7000418

[B7] Lee Y. Virgala I. Sadati S. M. H. Falotico E. (2024). Design, modeling and control of kinematically redundant robots. Front. Robotics And Ai 11, 1399217. 10.3389/frobt.2024.1399217 38651052 PMC11033426

[B8] Li S. Triolo R. J. Charkhkar H. (2025). A sensory neuroprosthesis enhances recovery from treadmill-induced stumbles for individuals with lower limb loss. Sci. Rep. 15, 1732. 10.1038/s41598-025-85788-4 39799143 PMC11724839

[B9] Meeseub L. Hyungbae P. Joonhong L. (2010). “Development of riding robot system for entertainment and healthcare service,” in Robotics and automation systems. Editors Brisan C. Maties V. Stan S. Brad S. , 253. 10.4028/www.scientific.net/SSP.166-167.253

[B10] Nguyen D. Hekman E. (2024). The news framing of artificial intelligence: a critical exploration of how media discourses make sense of automation. Ai & Soc. 39, 437–451. 10.1007/s00146-022-01511-1

[B11] Qu C. Wu B. Chen H. Yu C. Shen F. (2017). A somatosensory controlled upper limb exoskeleton mirror rehabilitation robot system. China Mech. Eng. 2, 2484–2489. 10.3969/j.issn.1004-132X.2018.20.014

[B12] Sobiech M. Wolanski W. Karpiel I. (2022). “Brief overview upper limb rehabilitation robots/devices,” in Digital interaction and machine intelligence. Editors Biele C. Kacprzyk J. Kopec W. Owsinski J. Romanowski A. Sikorski M. , 286–297. 10.1007/978-3-031-11432-8_29

[B13] Wang H. Zhang B. (2019). Application analysis of a new tilt coordination body sensing algorithm in flight simulators. Chongqing: Chongqing University Journal Press. 10.11835/j.issn.1000-582X.2019.05.003

[B14] Wu J. Liu Y. Zhao J. Zang X. Guan Y. (2022). Research on theory and a performance analysis of an innovative rehabilitation robot. Sensors 22, 3929. 10.3390/s22103929 35632338 PMC9147418

[B15] Wu J. Liu Y. Zhao J. Jia Z. (2023). Research on a new rehabilitation robot for balance disorders. Ieee Trans. Neural Syst. And Rehabilitation Eng. 31, 3927–3936. 10.1109/TNSRE.2023.3312692 37676800

[B16] Wu J. Liu Y. Chu G. Cai H. Li Y. Zhao J. (2024). Research on the configuration of a balanced disability rehabilitation robot based on redundant degrees of freedom. Adv. Mech. Eng. 16. 10.1177/16878132241272231

[B17] Zhang Z. Xu Z. (2017). Research on wa for three degree of freedom simulation motion platform. Mech. Sci. Technol. 10.13433/j.cnki.1003-8728.2018.0324

[B18] Zhang X. Chen L. Dong W. Li C. (2024). Optimizing redundant robot kinematics and motion planning via advanced d-h analysis and enhanced artificial potential fields. Electronics 13, 3304. 10.3390/electronics13163304

[B19] Zhang Y. Zhang D. Wu F. Ton P. (2024). Analysis of the comprehensive treatment effect of traditional Chinese medicine with internal and external characteristics after meniscus injury surgery. Zhejiang J. Traditional Chin. Med. 59. 10.13633/j.cnki.zjtcm.2024.08.026

